# High-Performance Sensor Based on Molecularly Imprinted Poly-o-Phenylenediamine for Determination of Pentoses in Hydrolyzates of Lignocellulosic Biomass

**DOI:** 10.1021/acsomega.5c08961

**Published:** 2026-02-27

**Authors:** Miguel Sales Porto de Sousa, Acelino Cardoso de Sá, João Pedro Jenson de Oliveira, Cristián A. Ferreti, María N. Kneeteman, Leonardo Lataro Paim

**Affiliations:** † 327024 São Paulo State University (Unesp), School of Engineering and Sciences, Rosana, Barrageiros Avenue 1881, Rosana, SP 19274-000, Brazil; ‡ Institute of Physics, São Carlos, USP − University of São Paulo, 400 Trabalhador São-Carlense Aveneu, São Carlos, SP 13566-590, Brazil; § School of Electrical and Computer Engineering, University of Campinas (UNICAMP), Av. Albert Einstein 400, Campinas, SP 13083-852, Brazil; ∥ Instituto de Química Aplicada del Litoral (IQAL), Santa Fe, Universidad Nacional del Litoral-CONICET, Santiago del Estero 2654, Santa Fe S3000, Argentina

## Abstract

Molecularly imprinted polymer (MIP) sensors were developed to determine d-xylose and d-arabinose in hydrolyzates of lignocellulosic biomass from sugarcane. Sensors were fabricated using molecularly imprinted poly-o-phenylenediamine anchored on composite substrates of functionalized multiwalled carbon nanotubes (FMWCNTs) electrodeposited onto a graphite/paraffin surface. The characterization of the modified electrodes and electrochemical analyses involved the utilization of spectroscopy and electroanalytical techniques such as Fourier-Transform Infrared Spectroscopy (FT-IR), Field Emission Gun Scanning Electron Microscopy (FEG-SEM), Cyclic Voltammetry (CV), Electrochemical Impedance Spectroscopy (EIS), and Differential Pulse Voltammetry (DPV). These techniques were employed to gain insight into the modified electrodes’ properties and behavior and perform detailed electrochemical analyses. In addition, the most critical parameters that directly affect the analytical performance of the electrochemical sensor have been optimized. The sensors showed a limit of detection (LOD) of 6.1 × 10^–12^ mol L^–1^ for d-xylose and 2.5 × 10^–12^ mol L^–1^ for d-arabinose, in the linear range of concentration from 1.0 × 10^–11^ mol L^–1^ to 1.0 × 10^–10^ mol L^–1^. The employed sensor effectively facilitated the quantification of d-xylose and d-arabinose within hydrolyzate samples derived from sugarcane bagasse.

## Graphical abstract

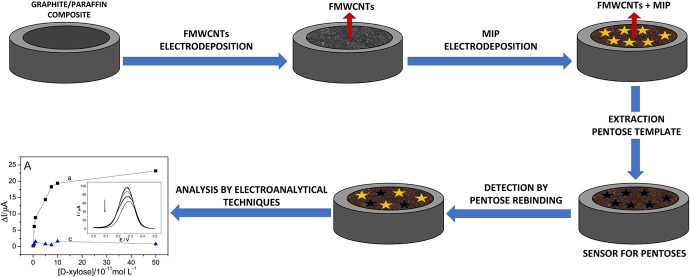

## 1 Introduction

The demand for renewable energy has driven the development and advancement of new technologies. Consequently, Brazil, the United States, and the European Union have significantly increased their efforts to produce second-generation (2G) ethanol, which offers a promising solution to the escalating energy crisis. 2G ethanol can alleviate the significant environmental damage resulting from overdependence on petroleum-based fuels.^1,2^

In recent years, the global production of bioethanol has markedly increased, aiming to transition production and energy systems toward more secure and environmentally sustainable pathways.^1,3,5^ Therefore, it can be posited that the energy derived from this resource has the potential to enhance societal energy security.^4^ Biomass utilization is characterized by notable attributes, including its ample accessibility and abundance, and the beneficial trait of not interfering with food resources.^5,6^ On average, each ton of cane processed for sugar and ethanol production yields 200 kg of straw and 250 kg of bagasse, with an approximate composition of 27–32% hemicellulose, 19–24% lignin, and 32–44% cellulose.^7,8^

Lignocellulosic biomass can be used for 2G ethanol production because cellulose and hemicellulose are composed of fermentable sugars, such as pentoses and hexoses.^9^ Hemicellulose is composed of xylose and arabinose (pentoses), mannose, glucose, and galactose (hexoses), as well as trace amounts of rhamnose, fucose, and acetyl groups.^10^ The conversion of pentoses into fermentable sugars is essential for the viability of 2G ethanol production, given the high concentration of structural pentoses in biomass.^9^

In Brazil, the potential for 2G ethanol is significant due to the plentiful availability of lignocellulosic biomass, such as sugarcane bagasse. Consequently, advancing research on the 2G ethanol production process is essential for its successful implementation.^11,12^ The production of 2G ethanol encompasses preprocessing, hydrolysis, and fermentation.^13^ During the hydrolysis phase, fermentable sugars are derived from cellulose and hemicellulose.^14^

In this context, it is imperative to comprehensively understand the chemical modifications that occur during the hydrolysis of lignocellulosic biomass to effectively manage and enhance the 2G ethanol process. One way to monitor the process is to use analytical tools that can determine the chemical composition of the hydrolyzates. Electrochemical techniques can provide real-time and in-process information. Furthermore, the use of electrochemical sensors during process steps allows system automation at a low cost, with fast detection and good sensitivity for analytes.^15^

Electrochemical sensors can be modified chemically using monolayers, thin films, or thick coatings.^16^ Chemically modified electrodes (CMEs) exhibit good selectivity and sensitivity to analytes of interest.^16,17^ For instance, CMEs modified with FMWCNTs exhibit good electrical properties, high sensitivity, chemical stability, and high surface area.^17,18^ The monitoring of carbohydrates, such as glucose, in complex biological fluids, such as saliva, requires sensors with exceptionally high specificity. The primary challenge in this field is the low selectivity of conventional sensors, which are susceptible to interference from other structurally similar molecules and coexisting substances within the matrix. Li et al.^18^ highlighted the significance of MIPs as a robust solution to this selectivity issue. The authors developed a biomimetic detection platform, specifically a paper-based microfluidic chip for visual detection, utilizing a “boronate affinity-oriented surface imprinting strategy” (BA-MIPs). The relevance of this approach lies in the synergy of two recognition mechanisms: the specific chemical affinity of boric acid for the cis-diol groups of the carbohydrate (glucose) and the conformational recognition (shape and size) provided by the MIP cavity. As demonstrated in this study, this combination facilitated the construction of recognition sites with “excellent selectivity” and “remarkable anti-interference capability”, effectively distinguishing glucose from other compounds.

Recently, our group published a work where we modified by electrodeposition of a graphite/paraffin composite electrode surface with FMWCNT.^19^ Surface modification of substrates by electrodeposition has some advantages, such as controlling the amount deposited and using small quantities of reagents.^20^ MIPs are a type of nanomaterial used to create CMEs.^21–23^ MIPs are synthetic polymers with high selectivity toward specific target molecules, have a low detection limit, and are easy to prepare, cost-effective, and reusable several times.^24,25^ The electropolymerization process used to create MIPs can be carried out directly on the surface of CMEs. The electrodes showed facile control over the thickness of the polymeric film and ensured stability.^21,25^

MIPs have emerged as a pivotal strategy for designing advanced chemical sensors. The primary significance of MIPs lies in their capacity to function as biomimetic receptors.^21^ As emphasized in the review by Kim and coauthors,^22^ MIPs present notable advantages, including high selectivity and sensitivity toward target molecules, superior physicochemical robustness, ease of synthesis, and cost-effectiveness. The molecular imprinting process facilitates the creation of cavities (binding sites) that are stereospecifically molded in size, shape, and arrangement of functional groups to selectively recognize the analyte of interest, emulating the “lock-and-key” mechanism observed in biological systems. This article examined the application of MIPs, encompassing both nonconducting polymers (MINPs) and conducting polymers (MICPs), illustrating their essential role in the development of selective and sensitive electrochemical sensors for various domains, such as environmental monitoring, clinical diagnostics, and food analysis.

The integration of the MIP technology with electrochemical sensors offers significant advantages. As reviewed by Shah et al.,^24^ the pivotal function of MIPs in this field attributed to their capacity to ensure selectivity, wherein the interaction between the analyte and the imprinted cavity of the polymer is directly translated into a quantifiable electrical signal, whether amperometric, potentiometric, or impedimetric. This review highlights the efficacy of this approach, illustrating that incorporating MIPs into the design of electrochemical sensors is essential for achieving an extremely low LOD, thereby enabling the quantification of analytes at picomolar or lower concentrations in complex matrices.

In this work, we developed composite sensors from graphite/paraffin substrates modified with FMWCNT by electrodeposition. This study aimed to analyze the presence of d-xylose and d-arabinose in hydrolyzates derived from sugarcane bagasse. To increase the selectivity of the sensors, molecularly imprinted polymers were formed from o-PD on the surface of composite electrodes with FMWCNT. The results show that the o-PD MIP electrode is an excellent alternative for the selective and sensitive detection of pentoses.

## 2 Experimental Section

### 2.1 Reagents and Chemicals

All reagents used in the experiments had high analytical purity and were used as received. The following chemicals were acquired from Synth: anhydrous d-glucose, potassium chloride, potassium ferrocyanide, and histological paraffin (∼58 °C). Sigma-Aldrich supplied the following chemicals: acetic acid anhydrous, d-arabinose (≥98.0%), d-xylose (≥98.0%), d-fructose (≥99.0%), d-sucrose (≥99.5%), o-phenylenediamine (99.5%), nitric acid (≥98.0%), dimethylformamide (DMF, 98.9%), and graphite powder (<20 μm). Sodium acetate (≥99.0%) was acquired from Dinâmica (Brazil). The probe solution consisted of 10.0 × 10^–3^ mol·L^–1^ K_3_[Fe­(CN)_6_] dissolved in 1.0 mol·L^–1^ KCl. The FMWCNTs stock solution was prepared in 0.55 mol·L^–1^ of HNO_3_. The electropolymerization stock solution contained a 3.0 × 10^–4^ mol·L^–1^ pentose template (d-xylose or d-arabinose) and 7.0 × 10^–3^ mol·L^–1^ o-PD in an acetate buffer solution with a pH value of 5.1. Deionized water was used throughout all experiments. A solution composed of a 5:2 volumetric ratio of *N*,*N*-dimethylformamide (DMF) to acetic acid was employed to extract the template molecules from the MIP matrix. The samples of sugarcane bagasse used in this study were sourced from the Alcohol and Sugar Industry located in the state of São Paulo, Brazil. The acid hydrolysis process was performed based on the laboratory protocols established by the National Renewable Energy Laboratory (NREL).^26^

### 2.2 Preparation of the Modified and Unmodified Electrodes

#### 2.2.1 Preparation of Graphite/Paraffin Substrates

The substrate was prepared by mixing graphite and paraffin in a 7:3 (v/v) ratio, analogously to the literature.^19,27–29^ This substrate was placed in the body of the insulin syringe (4.0 mm in diameter), connecting to the copper wire. For electrochemical behavior studies, the electrodes were kept at rest for 24 h for cooling and curing and then polished with abrasive paper.

#### 2.2.2 Modification of the Surface of the Electrode

The graphite/paraffin composite electrodes (GPEs) were modified through a two-step process using CV. In the first step, the electrode surfaces were functionalized with FMWCNTs, followed by subsequent modification with MIPs. The FMWCNTs were electrodeposited onto the electrode surface by cycling the potential from −0.5 to 1.0 V for 15 consecutive scans at a scan rate of 50 mV s^–1^ in the stock solution, following previously reported procedures.^30,31^ In sequence, the electropolymerization of MIPs was carried out between −0.4 and 1.0 V (50 mV s^–1^) for 20 consecutive cycles in acetate buffer solution (pH 5.1) containing 7.0 × 10^–3^ mol L^–1^ o-PD and 3.0 × 10^–4^ mol L^–1^ of each template molecule (d-xylose or d-arabinose), similarly to the literature.^32^ A nonimprinted polymer (NIP) was also synthesized under identical conditions but without the template molecule, to serve as a control for comparative analysis. After electropolymerization, the electrodes were washed for 60 s with a dimethylformamide and acetic acid mixture (5:2 v/v) to remove the template molecules. This solvent treatment disrupts the hydrogen bonds between the polymer matrix and the pentose (xylose or arabinose), enabling the release of imprinted molecules. Consequently, when the sensor is later immersed in a solution containing the target analyte, the specific rebinding process occurs (Figure 1).

**Figure 1:**
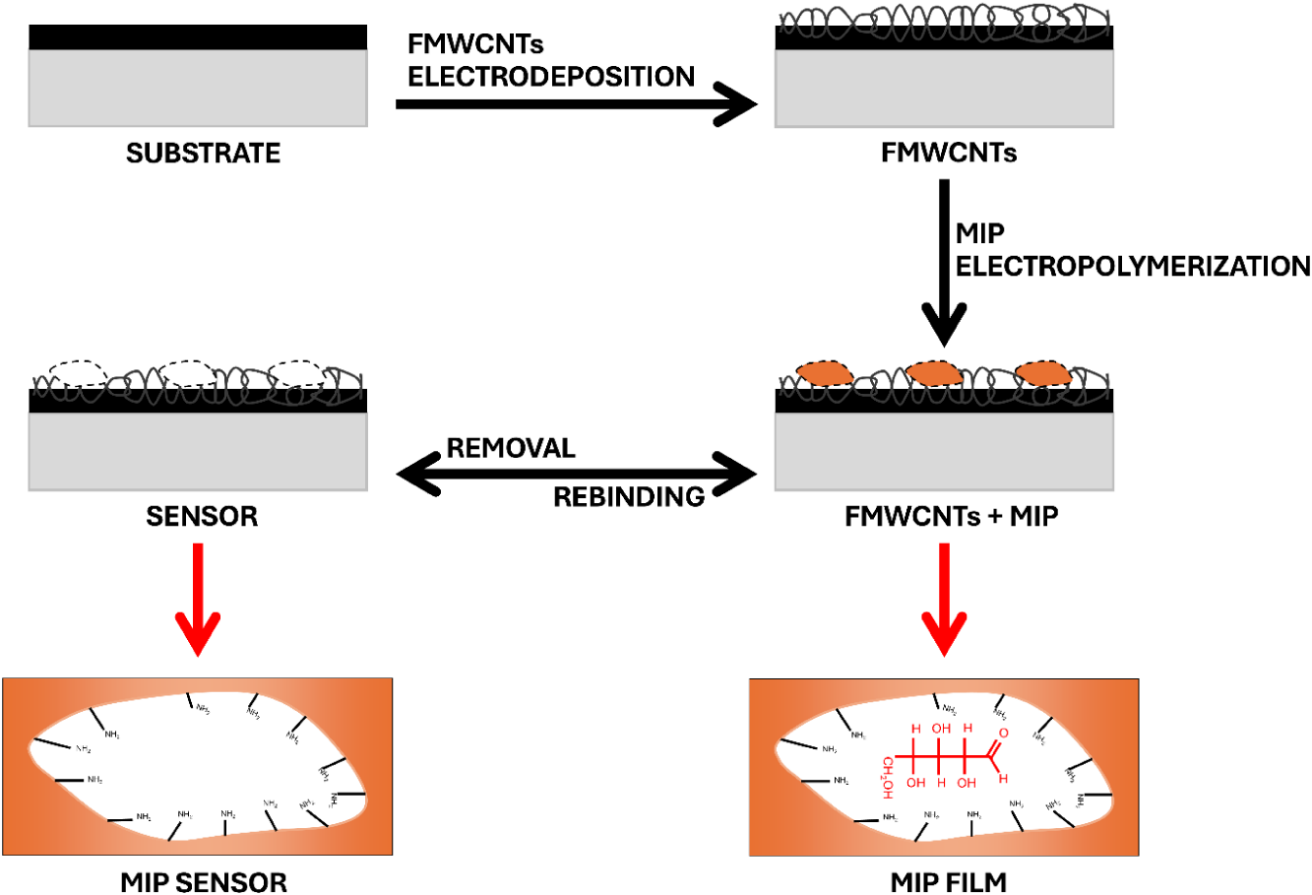
A scheme for manufacturing a working electrode modified with FMWCNTs and MIP.

### 2.3 Morphological Characterization

The microscopy characterization of the surface of electrodes modified with FMWCNTs and MIP was performed with a FEG-SEM model JSM-7500F from JEOL. The analyses were carried out by using voltages of 2–20 kV and a working distance between 4.0 and 8.5 mm. FT-IR analysis was employed to investigate the chemical composition of materials with an IRPrestige-21 instrument from Shimadzu. Pellets were prepared by combining 1% of the samples with the KBr mass. The spectral analysis was performed in the region between 400 and 4000 cm^–1^, with a resolution of 4 cm^–1^, and encompassing 32 replicates per analysis.

### 2.4 Sample Preparation

The raw material utilized in this study was sourced from the sugar and alcohol industry in Rosana, São Paulo State, Brazil. It was prepared following the laboratory protocols established by the NREL.^26^ First, the lignocellulosic material was dried and milled, and fractions of particles smaller than 0.5 mm were selected. Then, before the sample hydrolysis, aqueous and ethanolic extraction processes were performed, leaving the material free of extractives. Thus, 300 ± 10 mg of biomass was mixed with 3.00 ± 0.01 mL of 72% (v/v) sulfuric acid and then incubated for 60 ± 5 min in a water bath at 30 ± 3 °C. The mixture was then diluted by adding 84.00 ± 0.04 mL of deionized water and sent to the autoclave. After decompression of the autoclave, quantitative filter paper was used to separate the liquid fraction.

### 2.5 Electrochemical Measurements

The electrochemical measurements were conducted under ambient conditions employing a Potentiostat/Galvanostat PGSTAT204 instrument (Autolab, Metrohm) connected to a microcomputer equipped with Nova 2.1 control software for data acquisition and storage. A conventional three-electrode setup was utilized, comprising a CME as the working electrode, a platinum electrode as the counter electrode, and Ag/AgCl (KCl = 3.0 mol L^–1^) as the reference electrode. Furthermore, a magnetic stirrer was employed to promote convective transport.

All electrochemical measurements were conducted by using the [Fe­(CN)_6_]^3‑/4–^ redox couple as a standard probe to evaluate the modified electrode surfaces. The sensing mechanism is based on the modulation of the charge-transfer resistance (*R*_ct_) or peak current (*I*_p_) of the probe, which is impeded by the MIP layer. The binding of the carbohydrate template is anticipated to augment this impediment, thereby generating an analytical signal that can be detected using the developed sensor. A high concentration of KCl (1.0 mol L^–1^) was used as the supporting electrolyte to minimize the solution resistance and ensure diffusion-controlled mass transport. CV was used to characterize the electrode surface and ascertain the formal potential of the probe within a potential range (−0.2 to 0.8 V) that fully encompassed the redox peaks. Electrochemical impedance spectroscopy (EIS) measurements, the primary technique for sensing, were conducted at this formal potential (approximately 0.25 V) as this potential offers the highest sensitivity to variations in *R*_ct_. Differential pulse voltammetry (DPV) was employed for quantitative analysis owing to its higher sensitivity and ability to discriminate against capacitive current; its parameters (modulation amplitude, time, and step) were optimized to achieve the maximum signal-to-noise ratio.

All electrochemical measurements were carried out in a 10.0 × 10^–3^ mol L^–1^ of K_3_[Fe­(CN)_6_] solution containing 1.0 mol L^–1^ of KCl. All measurements were conducted at room temperature (25 °C). CVs were recorded by sweeping the potential between −0.2 and 0.8 V at a scan rate of 50 mV s^–1^. EIS analysis was performed at a fixed potential of 0.25 V over a frequency range of 0.1 Hz to 100 kHz. DPVs were recorded with a modulation time of 50 ms, a potential range of 0 to +0.5 V, a step potential of 5.0 mV, and a modulation amplitude of 25 mV.

## 3 Results and Discussion

### 3.1 Morphological Characterization

The surface of the GPE electrode (Figure 2(A)) observed by SEM spectroscopy exhibited a flat morphology with some roughness. On the other hand, the surfaces of the electrodes modified with FMWCNTs (Figure 2(B) and (C)) showed a significant amount of nanotubes. The FMWCNTs were observed at different magnifications. Modifying the sensor interface leads to a substantial increase in the number of active binding sites, enhances the efficiency of electron transfer, and imparts superior catalytic and conductive attributes. Consequently, the sensor exhibits heightened sensitivity regarding voltammetric response.^33^ Furthermore, the size of the electrodeposited nanoparticles was verified by the accomplishment of 388 measurements, ranging from 621 to 27 nm. The surface of the GPE/FMWCNTs modified with electropolymerized MIPs exhibited interesting features, with a greater aggregation of nanotubes observed, indicating a certain densification of the polymer layer (Figure 2(D)). This interesting feature is due to the formation of a complex between the o-PD functional monomer and the model molecule. In this way, binding sites adapted to the matrix are established, assigning an affinity condition for the target of analytical interest to the polymer. Figure S1 shows a schematic of the o-PD electropolymerization reaction, which illustrates the oxidative polymerization of diamine.^32^

**Figure 2:**
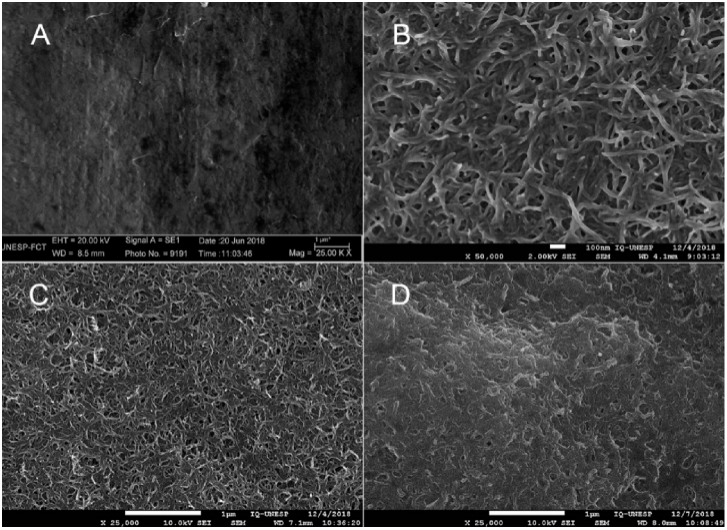
SEM images showing the surface morphology of the GPE electrode at 25000× (A) and the FMWCNTs/GPE electrode at 25000× (B) and 50000× (C) magnification and the MIP/FMWCNTs/GPE electrode at 25000× (D).

### 3.2 FTIR Characterization

Table 1 and Figure S11 show the results obtained by the FTIR technique for FMWCNTs, o-PD, d-xylose, d-arabinose, and MIP spectra. The FTIR spectra of FMWCNTs exhibit distinct peaks within specific wavenumber ranges. The observed peaks within the 2918–2850 cm^–1^ wavenumber range can be attributed to C–H elongation vibrations. Furthermore, the peak at approximately 1729 cm^–1^ corresponds to C–H elongation vibrations in conjunction with the carbonyl functional group. In addition, the peak at 3437 cm^–1^ can be ascribed to the O–H elongation vibrations. The range between 1698 and 1580 cm^–1^ encompasses peaks associated with the C=O and C=C functional groups in aromatic rings. The peaks around 1559 cm^–1^ also indicate elongation of the C=C bonds within the benzene ring. Lastly, the peaks around 1549 cm^–1^ can be attributed to the C–C elongation vibrations, possibly stemming from aromatic and unsaturated structures within this bond.^34,35^ Peaks around 1418 cm^–1^ are attributed to O–H plane deformation due to absorbed water, vibrations between peaks 1307 and 1061 cm^–1^ indicate C–O, peaks around 1256 cm^–1^ are attributed to C–O elongation, peaks around 1061 cm^–1^ are attributed to flat deformation, and peaks around 667 cm^–1^ can be attributed to C–H elongation.^36,37^

**Table 1:** Attribution of Functional Groups Associated with Significant Vibration Bands in the FTIR Spectra of FMWCNTs, o-PD, d-Xylose, d-Arabinose, and MIPs

FMWCNTs	3456 cm^–1^ (O–H), 2918–2850 cm^–1^ (C–H), 1729 cm^–1^(carbonyl functional group), 1698–1580 cm^–1^ (C=O and C=C),1559 cm^–1^ (C–C; benzene ring), 1549 cm^–1^ (C–C), 1418 cm^–1^ (O–H), 1307–1061 cm^–1^(C–O), 667 cm^–1^ (C–H)
o-PD	3445–3026 cm^–1^ (NH_2_), 1682–1589 cm^–1^ (C=C), 1559–1540 cm^–1^ (NH_2_), 1517–1423 cm^–1^ (C=C), 1274–1116 cm^–1^ (C–N), 871–710 cm^–1^ (C–H)
Arabinose	3379 cm^–1^ (O–H), 2929 cm^–1^ (C–H), 2689 cm^–1^ (C–H; aldehyde), 1730–1716 cm^–1^ (C=O), 1459–1437 cm^–1^ (CH_2_), 1410 cm^–1^ (CH_2_), 1136–999 cm^–1^ (C–O)
Xylose	3442 cm^–1^ (O–H), 2924–2893 cm^–1^ (C–H), 1796–1699 cm^–1^ (C=O), 1437–1460 cm^–1^ (CH_2_), 1112–1047 cm^–1^ (C–O)
MIP	3453 cm^–1^ (O–H/NH_2_), 2913–2851 cm^–1^ (C=H), 1738–1719 cm^–1^ (C=O), 1696–1593 cm^–1^ (C=O/C=C), 1579–1568 cm^–1^ (C=C), 1558–1539 cm^–1^ (NH_2_/C=C), 1520 cm^–1^ (NH_2_), 1506 cm^–1^ (C=C), 1487–1477 cm^–1^ (CH_2_), 1470–1437 cm^–1^ (C=C/CH_2_), 1420 cm^–1^ (CH_2_), 1387–1360 cm^–1^ (C–H), 785–717 cm^–1^ (C–H), 668 cm^–1^ (C–H)

The o-PD monomer demonstrates specific vibrational patterns within its molecular structure. These vibrations can be observed in various regions of the infrared spectrum. Notably, primary amines exhibit elongation vibrations between 3445 and 3026 cm^–1^ peaks. A peak at 1682 cm^–1^ corresponds to the stretching of the C=C bond, while peaks in the range 1589 cm^–1^ are attributed to the aromatic stretching of the C=C bond. Additionally, vibrations within the 1559–1540 cm^–1^ range indicate stretching of the NH_2_ group. Peaks in the 1517–1423 cm^–1^ range are associated with stretching of the C=C bond, while vibrations between 1274 and 1116 cm^–1^ represent elongation of the C–N bond. Furthermore, 871–710 cm^–1^ peaks indicate the vigorous intensity of aromatic C–H elongation vibrations resulting from the folding out of the phenazine ring plane.^38^

For d-arabinose, the peak at 3379 cm^–1^ was attributed to the elongation vibration of O–H, the 2929 cm^–1^ peak was associated with the C–H stretching vibration of the aliphatic functional group, the 2689 cm^–1^ peak was associated with the aldehyde C–H functional group, C–H stretching vibrations of the aldehyde functional group are in the range of 1730–1716 cm^–1^, peaks between 1459 and 1437 cm^–1^ are associated with CH_2_ elongation, the 1410 cm^–1^ peak was associated with CH_2_ elongation adjacent to the carbonyl group, and peaks between 1136 and 999 cm^–1^ are associated with C–O elongation. On the other hand, for d-xylose, the peak 3442 cm^–1^ is attributed to the elongation vibration O–H, peaks between 2924 and 2893 cm^–1^ are associated with the elongation of the C–H aliphatic functional group, peaks in the range 1796–1699 cm^–1^ are associated with C=O elongation of the aldehyde functional group, the peak between 1437 and 1460 cm^–1^ is associated with CH_2_ elongation, and peaks between 1112–1047 cm^–1^ are associated with C–O elongation.

In the MIPs, superposition was observed at some peaks, mainly because of the modification previously performed with FMWCNTs on the electrochemical sensor surface. The recorded peaks showed the same functional groups using d-xylose and d-arabinose as template molecules. Thus, there is a record of the 3453 cm^–1^ peak attributed to O–H or NH_2_ elongation, peaks between 2913 and 2851 cm^–1^ associated with stretching C=H, peaks between 1696 and 1593 cm^–1^ associated with stretching C=O and C=C, peaks between 1696 and 1593 cm^–1^ associated with stretching C=C, peaks in the range 1696–1593 cm^–1^ associated with elongation NH_2_ and C=C, peak 1520 cm^–1^ attributed to NH_2_ elongation, peak 1506 cm^–1^ associated with stretching C=C, peaks between 1487 and 1477 cm^–1^ associated with CH_2_ elongation, peaks in the range 1470–1437 cm^–1^ associated with elongation of C=C and CH_2_, peak 1420 cm^–1^ attributed to CH_2_ elongation, peaks in the range 1387– 1360 cm^–1^ associated with CH elongation, and peaks between 785 and 717 cm^–1^ associated with CH elongation.

### 3.3 Electrochemical Characterization

The electrochemical characterization of the chemically modified electrode was performed using DPV, EIS, and CV. The DPV analysis in Figure 3 (A) and (B) represents the behavior of the sensors after modifications were made on their surfaces, where a remarkable similarity was found among the voltammograms recorded for d-xylose (Figure 4A) and d-arabinose (Figure 4B). Curve (a) shows the behavior of the electrode after polishing. After the electrodeposition of FMWCNTs, a significant increase in the faradaic current was observed in curve (b) owing to better conductivity. Finally, in curve (c), the effect of electropolymerization on 20 consecutive cyclic scans is recorded, where the formation of a nonconductive polymer film for both pentoses is perceptive due to the drastic current reduction.

**Figure 3:**
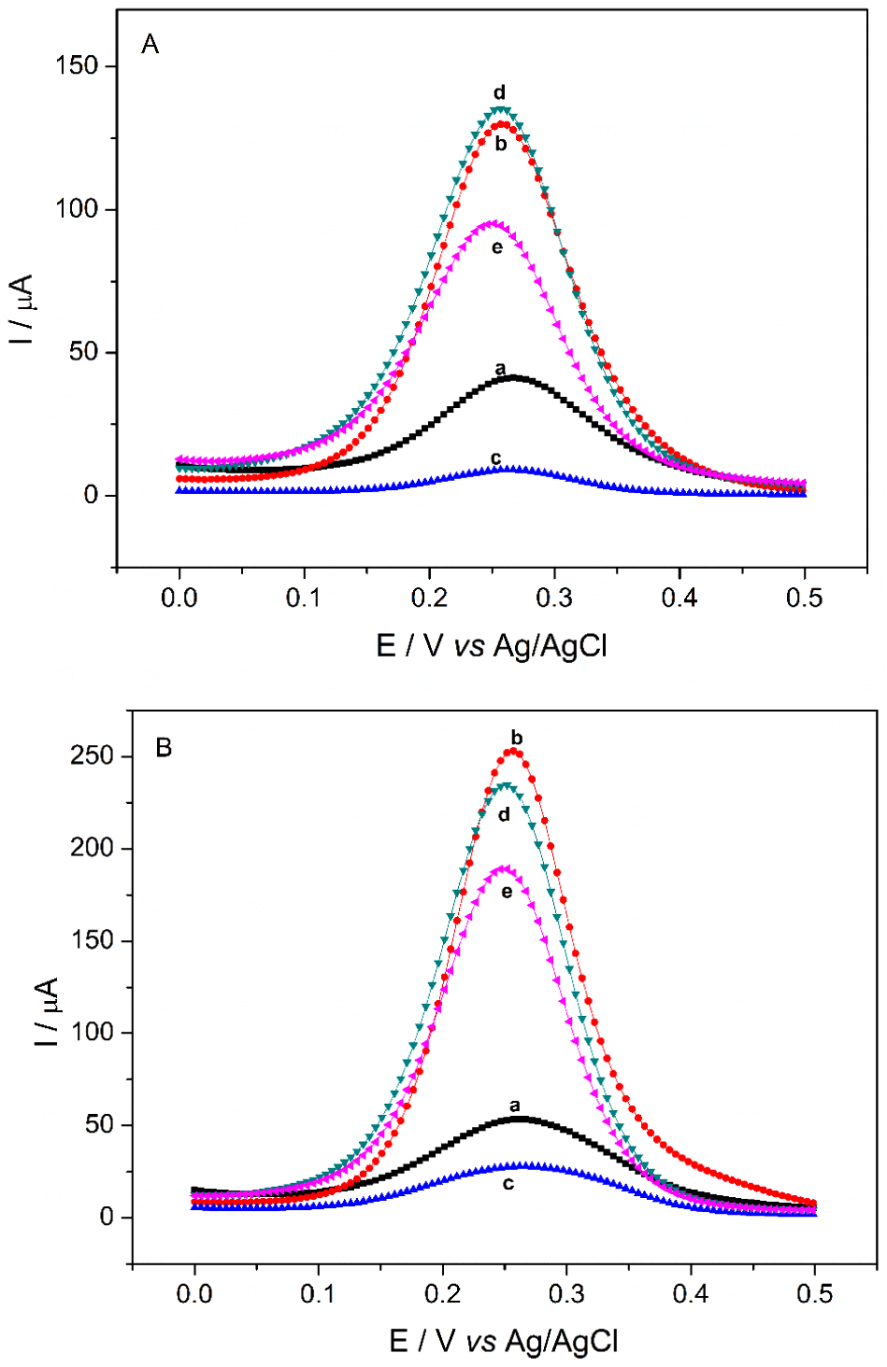
DPVs in 10.0 × 10^–3^ mol L^–1^ K_3_[Fe­(CN)_6_] in 1.0 mol L^–1^ KCl for d-xylose (A) and d-arabinose (B): GPE without modification (a), after the electrodeposition of FMWCNTs (b), FMWCNTs/GPE after electropolymerization (c), MIP/FMWCNTs/GPE after removal of pentoses (d), and MIP/FMWCNTs/GPE after the adsorption process in pentoses (e).

**Figure 4:**
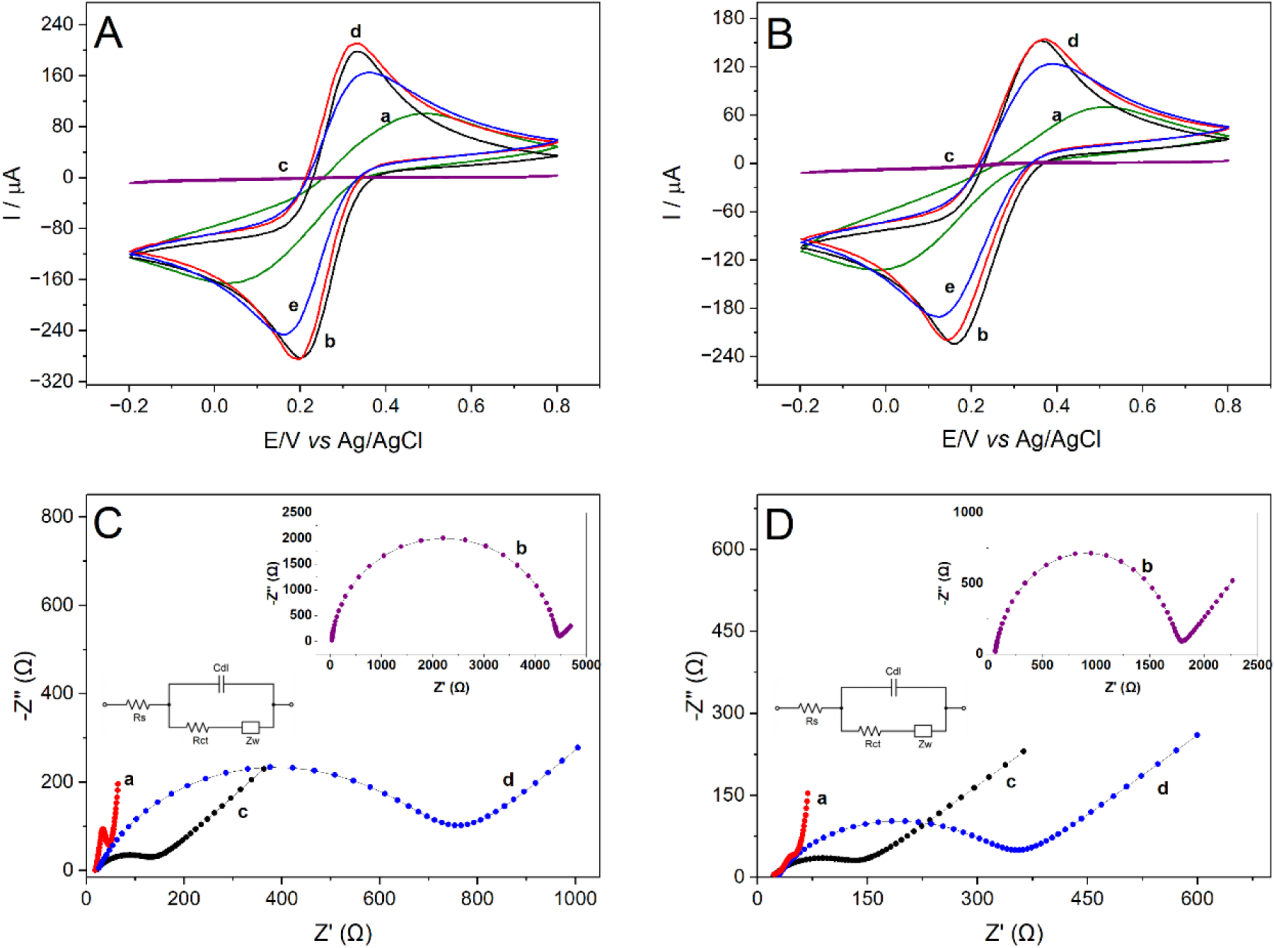
**(A, B)** CV spectra in 10.0 × 10^–3^ mol L^–1^ K_3_[Fe­(CN)_6_] in 1.0 mol L^–1^ KCl of the GPE without modification (a), after the electrodeposition of FMWCNTs (b), FMWCNTs/GPE after electropolymerization with d-xylose (A-c), FMWCNTs/GPE after electropolymerization with d-arabinose (B-c), Xil-MIP/FMWCNTs/GPE after removal of d-xylose (A-d), Ara-MIP/FMWCNTs/GPE after removal of d-arabinose (B-d), Xil-MIP/FMWCNTs/GPE after the adsorption process in 7.5 × 10^–8^ mol L^–1^ d-xylose (A-e), and Ara-MIP/FMWCNTs/GPE after the adsorption process in 7.5 × 10^–8^ mol L^–1^ d-arabinose (B-e). (C) EIS spectra of the Xil-MIP/FMWCNTs/GPE and (D) Ara-MIP/FMWCNTs/GPE after (a) the electrodeposition of FMWCNTs, (b) electropolymerization with templates, (c) removal of templates, and (d) the adsorption process in 7.5 × 10^–8^ mol L^–1^ of templates (d). Insets in C and D show the equivalent circuit for MIP electrodes.

For comparison purposes, electropolymerization with the template molecule (d-xylose or d-arabinose) and without the template molecule is shown in Figure S2 A, B, and C. The difference between electropolymerization in the presence and absence of pentose was not identified. This investigation establishes that within the potential range of electropolymerization, d-xylose and d-arabinose molecules exhibit no discernible oxidation or electrochemical reduction. In curve (d), a notable increase in the current was observed after the extraction phase of the template. This increase can be attributed to the cavities on the electrode surface. Curve (e), however, demonstrates a reduction in the peak current, which can be explained by the occupancy of these cavities with the target analyte molecules, displacing the template molecules.

The use of graphite and paraffin in the composition of the electrode aims to simultaneously promote sensitivity and robustness. So, through the electrodeposition of FMWCNTs, a significant interaction on the surface of the electrode is sought, justified by the susceptible electrical properties of this material.^39^ The cyclic voltammograms (CVs) depicted in Figure 4A and B illustrate the variations in the voltammetric profiles at each stage. Notably, there was an increase in the anodic and cathodic peak currents and a reduction in Δ*E*_p_ (|*E*_pa_ – *E*_pc_|) from 460 to 128 mV, which was attributed to the electrodeposition of FMWCNT onto the GPE surface. Following the formation of the molecularly imprinted polymers (MIPs), a nonconductive film developed on the C/FMWCNT surface, leading to the disappearance of the voltammetric profile, as evidenced by the voltammograms in Figure 4A­(c) and B­(c). A decline in peak currents and an increase in Δ*E*_p_, attributed to the filling of cavities by template molecules, are observed when comparing the voltammograms in Figure 4A and B after the extraction of template molecules (d) and the subsequent rebinding process (e). For Xil-MIP/FMWCNTs/GPE, the Δ*E*_p_ value increased from 135 to 198 mV postrebinding, whereas for Ara-MIP/FMWCNTs/GPE, the Δ*E*_p_ value increased from 195 to 254 mV.

An investigation into the alterations in the impedance occurring at the surface of the electrode was carried out by using the EIS method. In the semicircular part, the diameter of the curve indicates the magnitude of the *R*_ct_.^24^ Figure 4 (C) and (D) shows the Nyquist spectra of the EIS, where similar behaviors are recorded for both pentoses, and the inset shows the equivalent circuit [*R*_s_([*R*_ct_ *Z*_w_]*C*_dl_)], where the variable *R*_s_ represents the resistance of the solution, *C*_dl_ is the constant phase element with double layer capacitance, *R*_ct_ is the charge transfer resistance, and *Z*_w_ is the Warburg impedance that reflects the diffusion of ionic species in the porous structure. As observed in spectra 4C (a) and 4D (a), the FMWCNTs/GPE electrodes show a high electron transfer rate with an *R*_ct_ value of 119.5 Ω. Spectra (b) show the behavior of the electrode surface after electropolymerization. An increase in *R*_ct_ was observed for values above 4379 and 1690 Ω for Xil-MIP/FMWCNTs/GPE and Ara-MIP/FMWCNTs/GPE, respectively, which implied the development of a polymeric film on the electrode surface. Spectra (c), on the other hand, present values for *R*_ct_ of 404 Ω for Xil-MIP/FMWCNTs/GPE and 142 Ω for Ara-MIP/FMWCNTs/GPE, indicating cavities obtained by extracting the model molecule. Moreover, in spectra 4C (d) and 4D (d), owing to the rebinding of pentoses in the printed cavities, an increase in *R*_ct_ of approximately 712 and 317 Ω was observed for Xil-MIP/FMWCNTs/GPE and Ara-MIP/FMWCNTs/GPE, respectively.

During MIP polymerization in the presence of the template, the dense polymer film impedes charge transfer, leading to a notable increase in *R*_ct_. The removal of the template results in the formation of specific cavities within the polymer, facilitating the passage of the redox mediator. Consequently, there was a marked reduction in *R*_ct_, as these cavities enhanced the film’s permeability. Upon reintroduction of the analyte (template), it occupies the MIP cavities, thereby obstructing the passage of the redox mediator to the electrode. As a result, *R*_ct_ increases in proportion to the amount of re-encapsulated analyte; this increase forms the basis for quantitative detection in impedimetric sensors and is fundamental to the analytical signal of the redox peak currents in voltammetric techniques.^18,22^

The influence of the scan rate on the MIP/FMWCNTs/GPE sensor was also verified. Based on the data presented in Figure 5, it can be observed that an increase in the scan rate led to a corresponding increase in the peak current. This behavior can be explained by the size of the diffuse layer, which decreases as the velocity increases, resulting in interactions with a lower hydration radius.^40^ Furthermore, for the anodic (*I*_pa_) and cathodic (*I*_pc_) currents, it was observed that both are proportional to the square root of the scanning velocity, which can be expressed as *I*_pa_ (μA) = 16.42345 v^1/2^ + 116.38141 (*R* = 0.9943) and *I*_pc_ (μA) = −17.71144 v^1/2^ – 110.81453 (*R* = 0.9931), thus indicating a diffusion-controlled system.^41^ This underscores that the observed diffusional behavior is anticipated as the sensor response is attributed to the ferricyanide probe rather than directly to the analyte.

**Figure 5:**
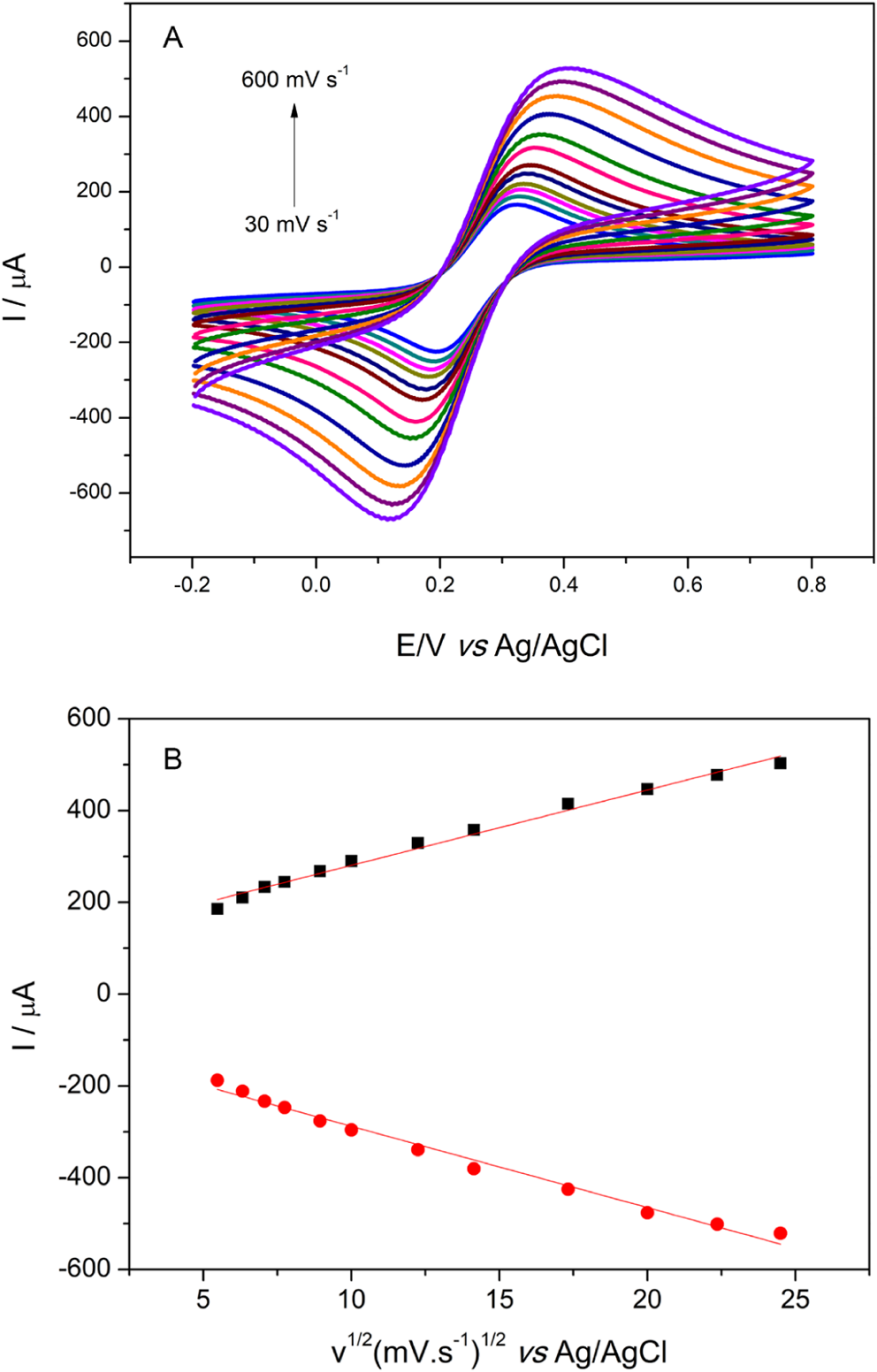
(A) CVs of MIP/FMWCNTs/GPE at different scan rates in 10.0 × 10^–3^ mol L^–1^ K_3_[Fe­(CN)_6_] in 1.0 mol L^–1^ KCl solution. (B) Influence of the scan rate on anodic and cathodic peak current vs square root of the scan rate (v ^1/2^).

### 3.4 Improvement of Experimental Parameters

The number of scanning cycles influences the thickness of the MIP film and, consequently, the sensitivity of the sensor.^42^ Therefore, a sequence of experiments in which the surface of the electrodes was modified with different numbers of cycles was performed to identify the best behavior. According to Figure S3, the number of 20 electropolymerization cycles is satisfactory because a considerable decrease in electrochemical performance is observed below 15 cycles, which can be attributed to the development of a thin polymer film. Furthermore, after 25 cycles, low behavior is again recorded owing to the development of a thick film that increases the resistance to mass transfer.

The pH used in electropolymerization has been optimized because the structure of the printed polymers and the target molecule can be influenced.^43^ Therefore, the electrodes were tested at pH values ranging from 4.3 to 5.3. As shown in Figure S4, pH 5.1 promoted the most significant interaction between the target molecule and the film. Moreover, at other pH values, the pentose molecules exhibited poor electrochemical oxidation behavior for the film.

The effect of the monomer concentration was verified in the electropolymerization process because it affects the polymer structure and rebinding affinity.^42^ Thus, to determine the appropriate amount of monomer, the o-PD concentration was varied from 3.0 × 10^–3^ mol L ^–1^ to 1.1 × 10 ^–2^ mol L^–1^. Figure S5 illustrates the challenges encountered in successfully generating printed cavities under low o-PD concentrations. The sensor exhibited a considerable decline in sensitivity below 5.0 × 10^–3^ mol L^–1^, indicating difficulty in forming effective cavities in the polymer matrix. Optimal performance was observed at an o-PD concentration of 7.0 × 10^–3^ mol L^–1^. Furthermore, at a concentration of 9.0 × 10^–3^ mol L^–1^ o-PD, the elimination of the model molecule was impeded due to extensive cross-linking.

The analysis focused on the influence of template molecule concentration because of its direct impact on the number of recognition cavities during the adsorption process.^44^ Figure S6 A and B depicts the impact of varying concentrations of d-xylose and d-arabinose, ranging from 1.0 × 10^–4^ mol L^–1^ to 9.0 × 10^–4^ mol L^–1^. For both pentoses, the best yield was obtained at a concentration of 3.0 × 1 x 10^–4^ mol L^–1^. At high concentrations, losses in selectivity were observed, probably due to the development of large cavities. On the other hand, the small amounts of cavities at low concentrations cause a lower sensitivity, as recorded for both pentoses.

The time required for the removal of the model molecule from the polymer film was optimized. This process allows the registration of cavities, characterized by a three-dimensional network that presents pores and the position of the functional groups, referring to the model.^45^ As shown in Figure S7 A, the sensor uses d-xylose as the model molecule. In Figure S7 B, the d-arabinose, after immersion of the sensors in the solution of C_3_H_7_NO and CH_3_COOH (5:2, v/v) under agitation, shows that after more than 45 s, the response begins to stabilize, and from 60 s, a saturation extraction time is obtained. A methanol solution with acetic acid was tested, but it was not as effective as the DMF/acetic acid mixture in extracting the target molecules. The influence of the adsorption time of the MIP was also analyzed, as shown in Figure S8 A and B. After 6 min, the response indicated a saturation adsorption time, suggesting that the printed locations could not be filled because they had already been combined with the target molecules.

### 3.5 Analytical Performance

#### 3.5.1 Analytical Curves

The optimized parameters were used for analytical curve studies. The Xil-MIP/FMWCNTs/GPE and Ara-MIP/FMWCNTs/GPE sensors were used at different concentrations of d-xylose and d-arabinose, respectively. Figure 6 (A) and (B) shows the current dependence of the pentose concentrations. The Δ*I* parameter was used to monitor the binding event between the analyte and specific recognition sites of the MIPs. The calculation was based on the absolute change in the peak current of the [Fe­(CN)­6]^3‑/4–^ redox probe, as shown in eq 1.

1 $$\Delta I=I_{\mathrm{p, probe}}-I_{\mathrm{p, pentose}}$$

**Figure 6:**
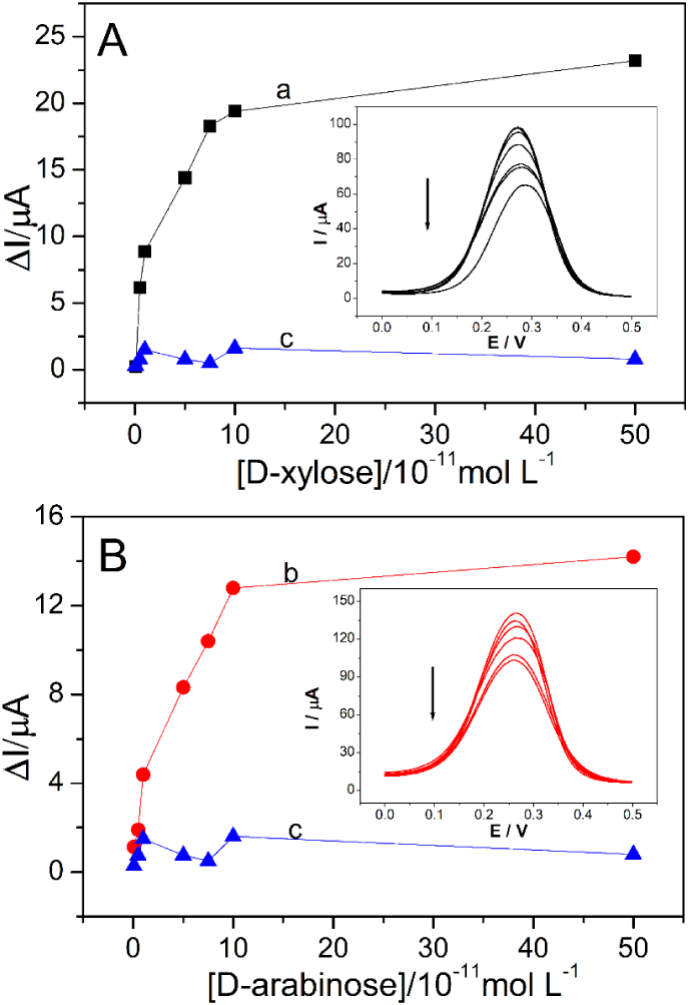
(A) Dependence of peak current variation (Δ*I*_p_) for concentrations of d-xylose on Xil-MIP/FMWCNTs/GPE (curve a) and NIP (curve c) sensors. (B) Dependence of Δ*I*_p_ for concentrations of d-arabinose on Ara-MIP/FMWCNTs/GPE (curve b) and NIP (curve c) sensors.

where *I*_p,probe_ is the peak current recorded for the empty MIP sensor (baseline) and *I*_p,pentose_ is the peak current recorded after incubation with a specific pentose concentration.

The Xil-MIP/FMWCNTs/GPE sensor showed Δ*I*_p_ equivalent to 8.86 μA in the 1 × 10^–11^ mol L^–1^ d-xylose solution, and an increase in Δ*I*_p_ was observed as the concentration increased. The same effect occurred for the Ara-MIP/FMWCNTs/GPE sensor, with Δ*I*_p_ equivalent to 4.39 μA for 1 × 10^–11^ mol L^–1^ d-arabinose. The effect of the concentration present in the MIP is justified by the filling of the remaining cavities after the extraction process of the model since the same does not occur for the NIP, characterized by the nonmolecularly imprinted polymer.

Within the concentration range of 1 × 10^–11^ mol L^–1^ to 1 × 10^–10^ mol L^–1^, a linear relationship was observed between the current and the concentrations of d-xylose and d-arabinose. The LOD for the MIPs electrodes was determined using established IUPAC-accepted methodologies.^46,47^ For d-xylose, a regression equation equivalent to Δ*I*_p_ = 1.38 × 10^5^·c + 7.53 × 10^–6^, with a regression coefficient (*R*) equal to 0.9970 and LOD = 6.07 × 10 ^–12^ mol L^–1^. The regression equation equivalent to Δ*I*_p_ = 9.54 × 10^4^·c + 3.45 × 10^–6^, *R* = 0.9988, and LOD = 2.54 × 10^–12^ mol L^–1^ was obtained for d-arabinose. Table 2 compares the results obtained for the Xil-MIP/FMWCNTs/GPE and Ara-MIP/FMWCNTs/GPE sensors with other techniques developed in the literature for sugar determination.

**Table 2:** The Linear Range and LOD of the Sensors Developed in This Study, Comparing Their Performance to That Reported in the Existing Literature

Electrode	Linear range (mol L^–1^)	LOD (mol L^–1^)	References
NiO/MWCNT	2.0 × 10^–4^ to 1.2 × 10^–2^	1.6 × 10^–4^	^48^
PDAP/RGO	5.0 × 10^–5^ to 6.5 × 10^–3^	1.4 × 10^–7^	^49^
rGO/(Ni–OH)_2_	2.0 × 10^–5^ to 3.0 × 10^–2^	1.5 × 10^–5^	^50^
CuOG-SPCE	1.2 × 10^–7^ to 5.0 × 10^–4^	3.43 × 10^–8^	^51^
Glu-PPD/GCE	2.5 × 10^–7^ to 2.5 × 10^–6^	1.8 × 10^–7^	^32^
CNT-CuNP hybrid paste electrode	1.0 × 10^–6^ to 2.0 × 10^–3^	1.8 × 10^–7^	^52^
NPsCu-GO/GCE	2.0 × 10^–5^ to 4.4 × 10^–4^	6.4 × 10^–6^	^53^
Xil-MIP/FMWCNTs/GPE	1.0 × 10^–11^ to 1.0 × 10^–10^	6.1 × 10^–12^	This work
Ara-MIP/FMWCNTs/GPE	1.0 × 10^–11^ to 1.0 × 10^–10^	2.5 × 10^–12^	This work

Thus, for all the sensors analyzed, the presence of two linear bands was verified, which can be justified by the levels of affinity between the template molecule and the cavities present on the surface of the electrode. To highlight the behavior of Xil-MIP/FMWCNTs/GPE and Ara-MIP/FMWCNTs/GPE, we found a low LOD for the other sensors. In assessing the affinity between d-xylose and d-arabinose and the designated sites, it can be posited that a strong affinity would result in a lower concentration of molecules occupying the cavities. Conversely, a weaker affinity necessitates a higher concentration of printed sites for adequate filling.

In this study, we explored the application of poly­(o-phenylenediamine) (PoPD) as a functional polymer in the development of molecularly imprinted polymers (MIPs) for the detection of pentoses, specifically d-xylose and d-arabinose, in lignocellulosic biomass hydrolyzates. The literature highlights the versatility of o-PD in various applications. For instance, the research conducted by Karimian et al.^54^ analyzed trace levels of the environmental pollutant perfluorooctanesulfonate (PFOS) in water, whereas Chuiprasert et al.^55^ focused on another emerging contaminant, the antibiotic ciprofloxacin. Furthermore, Abo-Elmagd et al.^56^ and Ting et al.^57^ extended the PoPD platform to the biomedical and clinical fields, targeting the cardioprotective drug cyclocreatine phosphate (CCrP) in the complex matrix of blood plasma and the cytokine Interleukin-6 (IL-6), respectively.

#### 3.5.2 Selectivity

The selectivity of the Xil-MIP/FMWCNTs/GPE and Ara-MIP/FMWCNTs/GPE sensors was analyzed by comparison with some possible interfering molecules with similar structures. Thus, the effect of d-glucose, d-fructose, and d-sucrose in determining d-xylose and d-arabinose is shown in Figure 7.

**Figure 7:**
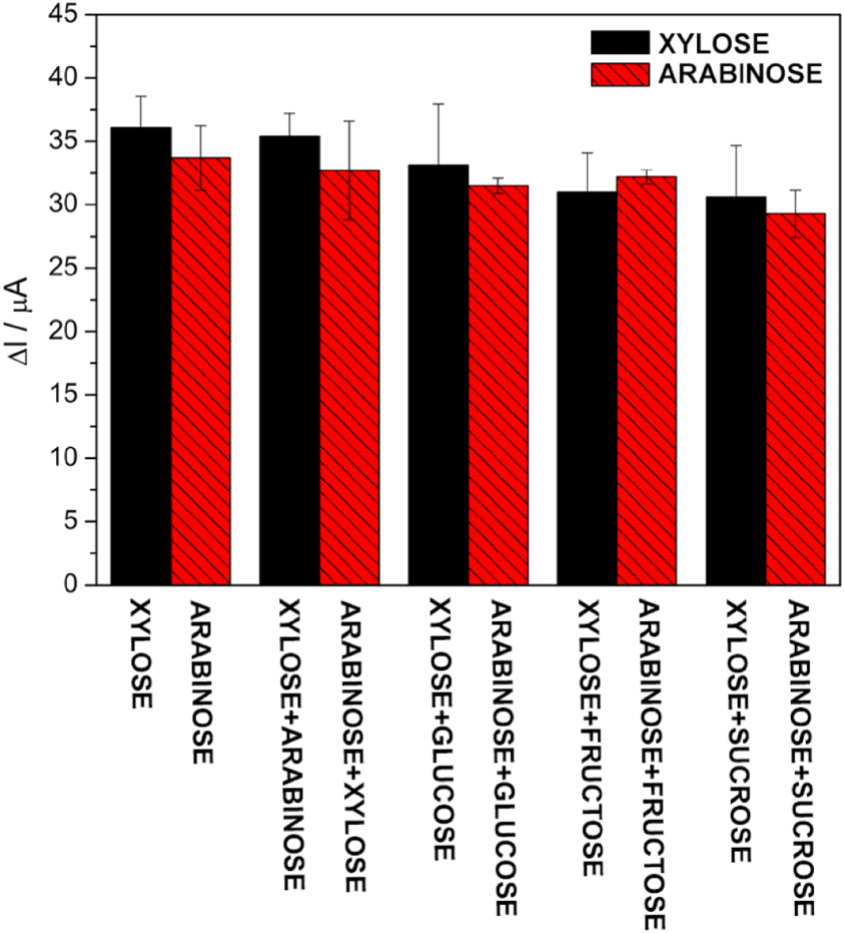
Black columns represent the behavior of Xil-MIP/FMWCNTs/GPE in the probe after adsorption in a solution containing 1 × 10^–11^ d-xylose, 1 × 10^–11^ d-xylose + 1 × 10^–10^ d-arabinose, 1 × 10^–11^ d-xylose + 1 × 10^–10^ d-glucose, 1 × 10^–11^ d-xylose + 1 × 10^–10^ d-fructose, and 1 × 10^–11^ d-xylose + 1 × 10^–10^ d-sucrose. At the same time, the red columns represent the behavior of Ara-MIP/FMWCNTs/GPE in the probe after adsorption in a solution containing 1 × 10^–11^ d-arabinose, 1 × 10^–11^ d-arabinose + 1 × 10^–10^ d-xylose, 1 × 10^–11^ d-arabinose + 1 × 10^–10^ d-glucose, 1 × 10^–11^ d-arabinose + 1 × 10^–10^ d-fructose, and 1 × 10^–11^ d-arabinose + 1 × 10^–10^ d-sucrose.

Because the sensor response is based on the interaction between the cavities left by the model and the studied molecule, it can perform a versatile approach with high selectivity.^21^ However, its measurement accuracy can be impaired by interfering agents, as seen by the size and functional groups of these molecules.^58^ The studies were performed by adding sugars that were ten times more concentrated than d-xylose and d-arabinose. In Figure S9 A and B, the effects of the concentration of 1 × 10^–10^ d-glucose, 1 × 10^–10^ d-fructose, and 1 × 10^–10^ d-sucrose in solutions of 1 × 10^–11^ d-xylose and 1 × 10^–11^ d-arabinose are shown.

#### 3.5.3 Reproducibility and Stability

Using the DPV technique, the reproducibility of the sensor was investigated using three different sensors that were prepared under the same conditions. Thus, after adsorption of 1 × 10^–11^ d-xylose in acetate buffer at pH 5.1, three times with the same sensor, its response was verified in a solution of 10.0 × 10^–3^ mol L^–1^ K_3_[Fe­(CN)_6_] in 1.0 mol L^–1^ KCl. The same sensor presented satisfactory reproducibility, with a relative standard deviation (RSD) of 2.92% for the currents determined at the pentose concentration. Furthermore, the three electrochemical sensors showed reproducibility with an RSD of 3.87% in response to a 1 × 10^–11^ d-xylose concentration. Therefore, it is plausible to state that the sensor has good reproducibility. The DPV technique was used again to observe that the electrochemical sensors presented a stable response. Thus, the sensor was stored for 19 days at room temperature, dried, and showed a response equivalent to 104% of the initial current, as shown in Figure S10.

### 3.6 Determination of Xylose and Arabinose in Real Samples

The lignocellulosic material obtained from the Rosana (State of São Paulo, Brazil) sugarcane industry, characterized in a similar way to the laboratory procedures of the National Laboratory of Renewable Energies,^26^ was used in a hydrolyzed state for the determination of pentoses. Considering the previous results, the Xil-MIP/FMWCNTs/CPE and Ara-MIP/FMWCNTs/CPE sensors exhibited good selectivity and sensitivity in the range of 1 × 10^–11^ to 1 × 10^–10^. The hydrolyzate was diluted ten million times. The reliability of the sensor was assessed in practical applications using the standard addition method, which has been documented in the literature.^32^ Various electrodes were used in the experimental setup. The obtained results highlight the promising potential of the developed sensors for accurately determining xylose and arabinose concentrations in industrial lignocellulosic samples. The outcomes of these experiments are presented in Table 3.

**Table 3:** Results Obtained for Pentoses in Hydrolyzed Samples of Sugarcane Bagasse

Pentose	Added (10^–11^ mol L^–1^)	Amount detected (10^–11^ mol L^–1^)	PeBias^a^ (%)	Recovery (%)
Arabinose	-	3.251 ± 0.047	-	-
	3.54	6.919 ± 0.190	3.47	103.6 ± 2.3
	5.31	8.171 ± 0.050	–7.94	92.6 ± 1.6
	7.08	9.887 ± 0.048	–6.69	93.7 ± 1.5
Xylose	-	0.847 ± 0.026	-	-
	3.00	3.977 ± 0.022	4.16	104.3 ± 3.2
	4.50	5.710 ± 0.034	7.47	108.1 ± 3.1
	6.00	6.628 ± 0.031	–3.78	96.4 ± 3.1

a PeBias: bias percent was calculated according to the literature.^46^

## 4 Conclusions

This study focuses on the development of composite graphite/paraffin sensors modified with FMWCNTs and MIPs. These sensors were designed to analyze and quantify d-xylose and d-arabinose, which are critical components of hydrolyzates derived from lignocellulosic biomass sourced from sugarcane. The sensor showed a LOD of 6.1 × 10^–12^ mol L^–1^ for d-xylose and 2.5 × 10^–12^ mol L^–1^ for d-arabinose in the linear range of 1.0 × 10^–11^ mol L^–1^ to 1.0 × 10^–10^ mol L^–1^. Furthermore, the sensor was successfully applied to determine d-xylose and d-arabinose in hydrolyzate samples, obtaining recovery values of 96.4–108.1% and 92.6–103.6%, respectively. The obtained results demonstrate the excellent practicality of these sensors for 2G ethanol production. In addition, the sensors exhibit notable attributes such as high sensitivity, excellent selectivity, reproducibility, and cost-effectiveness. These characteristics make them highly suitable for deployment in 2G ethanol production.

## Supplementary Material



## Notes

Electropolymerization reaction of o-PD; cyclic voltammetry of electropolymerization of o-PD in the presence of d-xylose and comparison of the last cycle of MIP and NIP; the number of cycles of deposition; effect of pH on the peak current; effect of the concentration of o-PD on peak current; effect of concentration of d-xylose and d-arabinose; effect of the extraction time of d-xylose and d-arabinose on peak current variation; effect of the rebinding time of d-xylose and d-arabinose on peak current variation; differential pulse voltammograms of sensor Xil-MIP/FMWCNTs/GPE with interference; differential pulse voltammograms for the sensor after some days; FTIR spectra for several steps of the sensor (Figures S1–S8) (PDF)
