# Supplementary material for: High-Performance Sensor Based on Molecularly Imprinted Poly-o-Phenylenediamine for Determination of Pentoses in Hydrolyzates of Lignocellulosic Biomass
Source: ACS Omega. 2026 Feb 27;11(9):14402–13. doi: 10.1021/acsomega.5c08961 (PMC12980417; doi:10.1021/acsomega.5c08961)
Supplement: Supplementary file 1 [file ao5c08961_si_001.pdf]

Supplementary Material

High-performance sensor based on molecularly imprinted poly-o-phenylenediamine for determination of pentoses in hydrolysates of lignocellulosic biomass.

Miguel Sales Porto de Sousa[1], *Acelino Cardoso de Sá*[2], *João Pedro Jenson de Oliveira*[3], *Cristián A. Ferreti*[4], *María N. Kneeteman*[4], *Leonardo Lataro Paim*[1]*.

[1] São Paulo State University (Unesp), School of Engineering and Sciences, Rosana, Barrageiros Avenue 1881, 19274-000, Rosana, SP, Brazil.

[2] Institute of Physics, São Carlos, USP – University of São Paulo, 400 Trabalhador São-Carlense Aveneu 13566-590, São Carlos, SP, Brazil.

[3] School of Electrical and Computer Engineering, University of Campinas (UNICAMP), Av. Albert Einstein 400, Campinas, SP 13083-852, Brazil.

[4] Instituto de Química Aplicada del Litoral (IQAL), Santa Fe, Universidad Nacional del Litoral - CONICET, Santiago del Estero 2654, S3000, Santa Fe, Argentina.

*E-mail: leonardo.paim@unesp.br

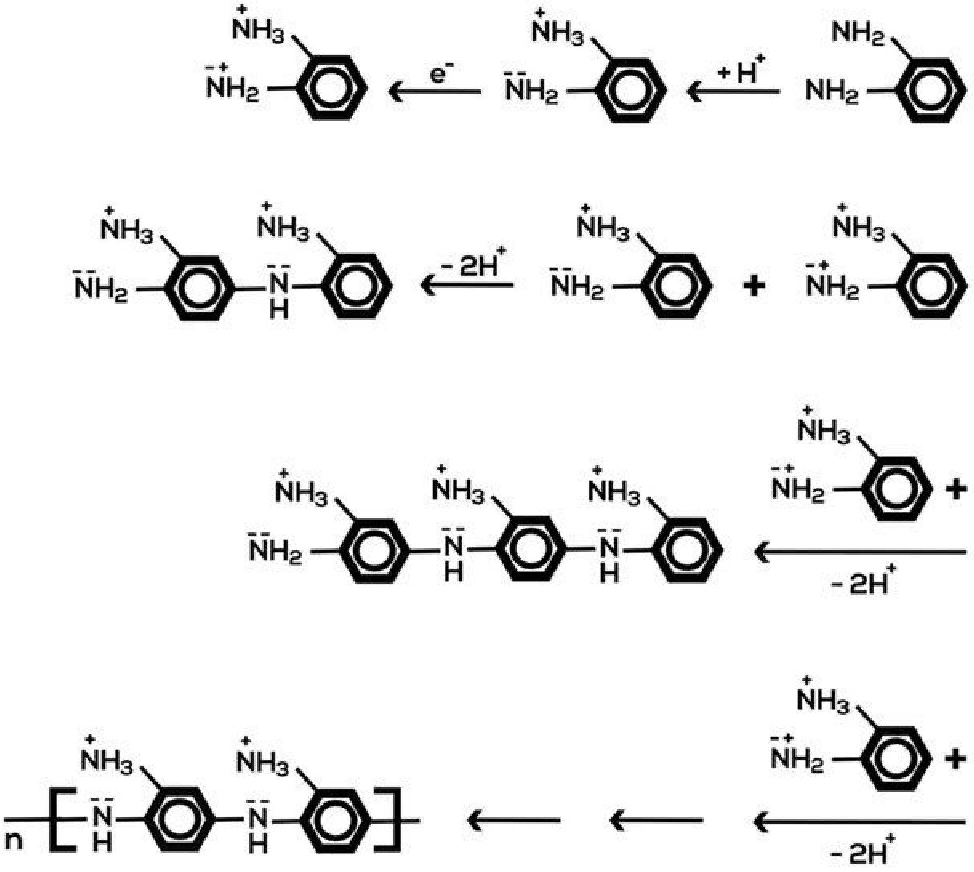

Figure S1. Electropolymerization reaction of o-Phenylenediamine (o-PD) [35].

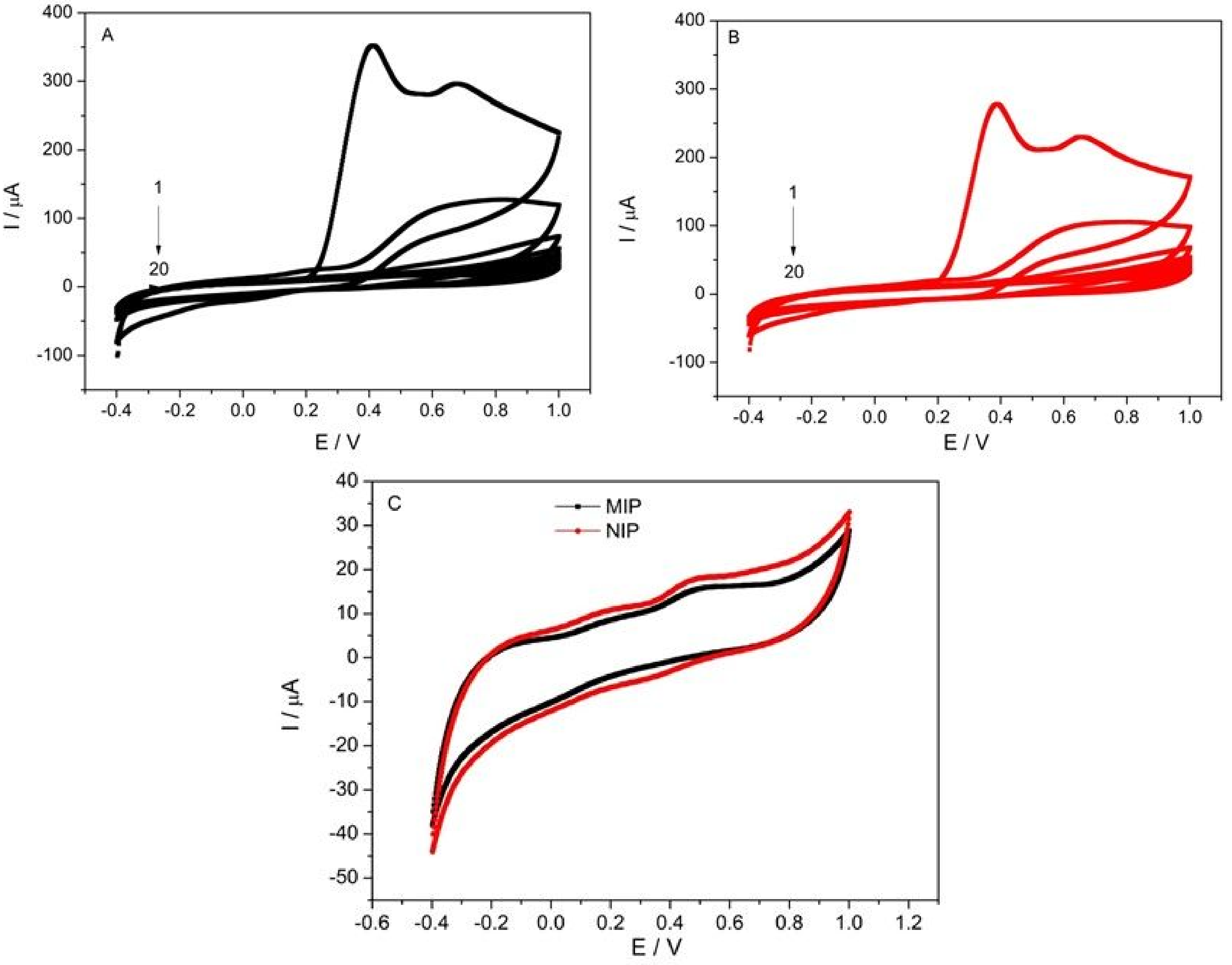

Figure S2. (A) Electropolymerization in 20 cycles in acetate buffer pH 5.1 with 7.0 x 10^{-3} mol L^{-1} o-PD in the presence of 3.0 x 10^{-4} mol L^{-1} D-xylose (MIP). (B) Electropolymerization in 20 cycles in acetate buffer pH 5.1 with 7.0 x 10^{-3} mol L^{-1} o-PD without model molecule (NIP). (C) Comparison of the last cycle of MIP and NIP during electropolymerization.

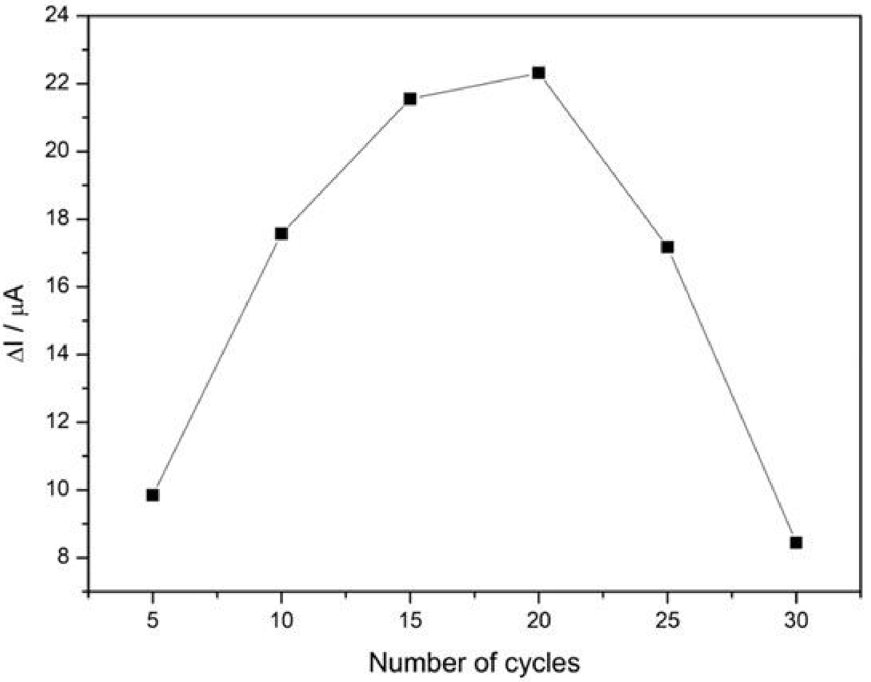

Figura S3. The number of cycles concerning peak current variation in the presence of $10x10^{-3}$ mol L^{-1} $K_3[Fe(CN)_6]$ in 1.0 mol L^{-1} KCl.

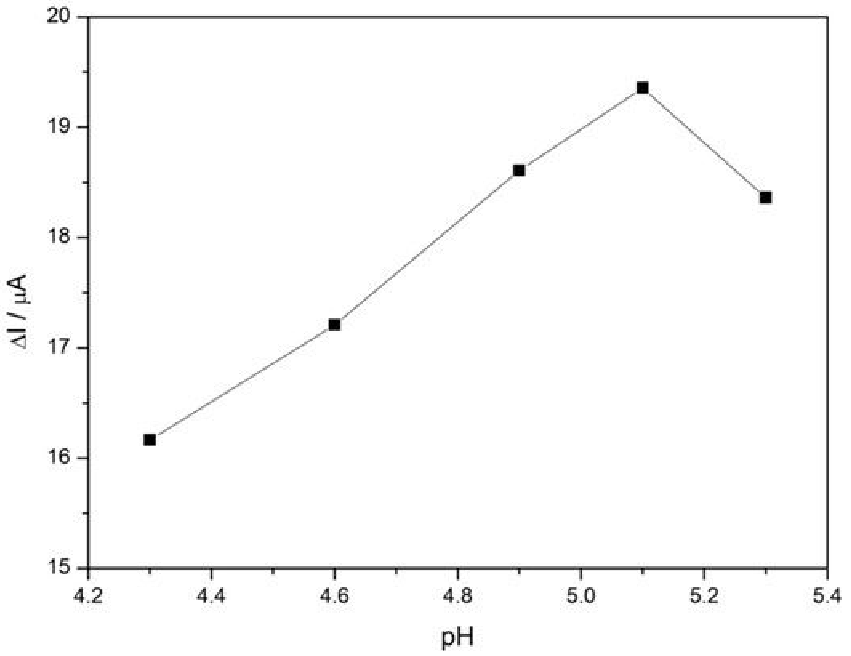

Figure S4. Effect of the pH value on the peak current at the concentration of 10.0 x 10^{-3} mol L^{-1} $K_3[Fe(CN)_6]$ in 1.0 mol L^{-1} KCl.

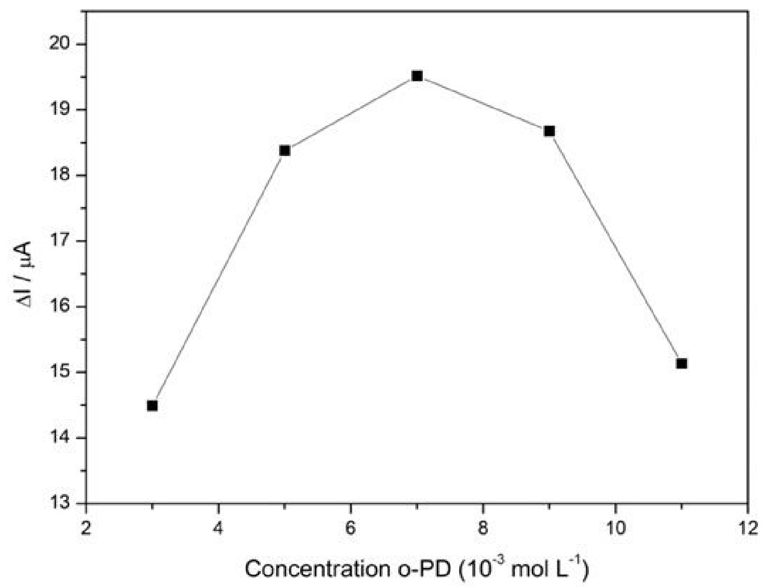

Figure S5. Effect of concentration o-PD (10^{-3} mol L^{-1}) on peak current variation at the concentration of 10.0 x 10^{-3} mol L^{-1} K_3 $[Fe(CN)_6]$ in 1.0 mol L^{-1} KCl.

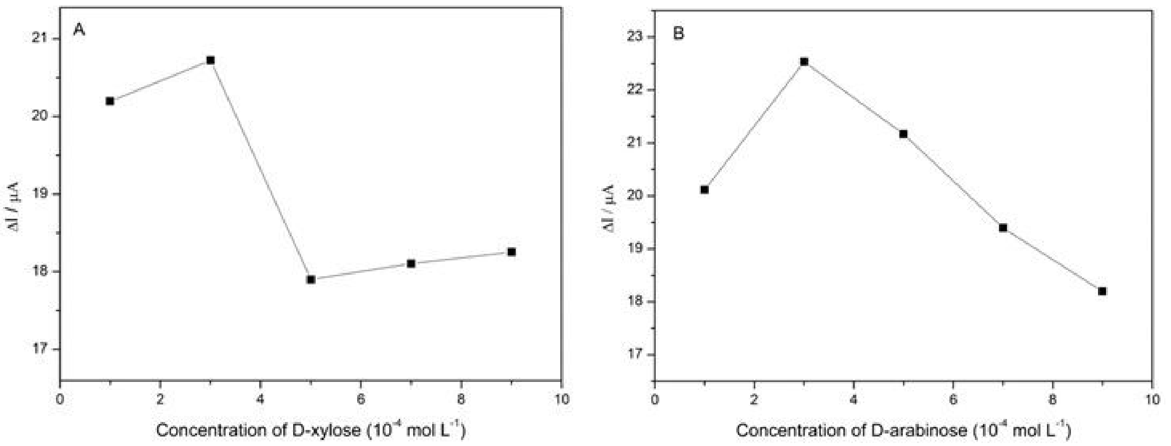

Figure S6. (A) Effect of concentration D-xylose (10^{-4} mol L^{-1}) and (B) D-arabinose (10^{-4} mol L^{-1}) on peak current variation at the concentration of 10.0 x 10^{-3} mol L^{-1} K_3 $[Fe(CN)_6]$ in 1.0 mol L^{-1} KCl.

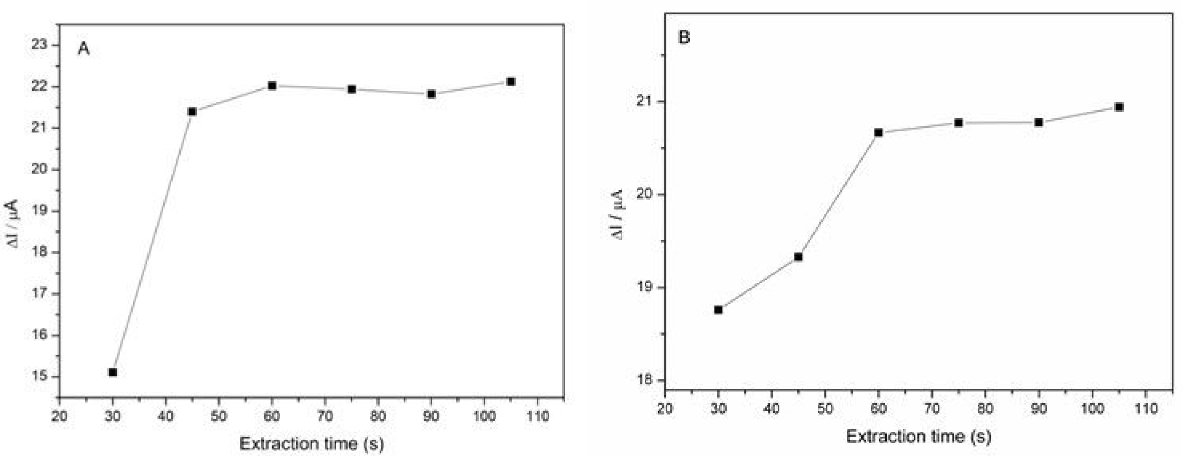

Figure S7. (A) Effect of the extraction time of D-xylose and (B) D-arabinose on peak current variation at the concentration of 10.0 x 10^{-3} mol L^{-1} $K_3[Fe(CN)_6]$ in 1.0 mol L^{-1} KCl.

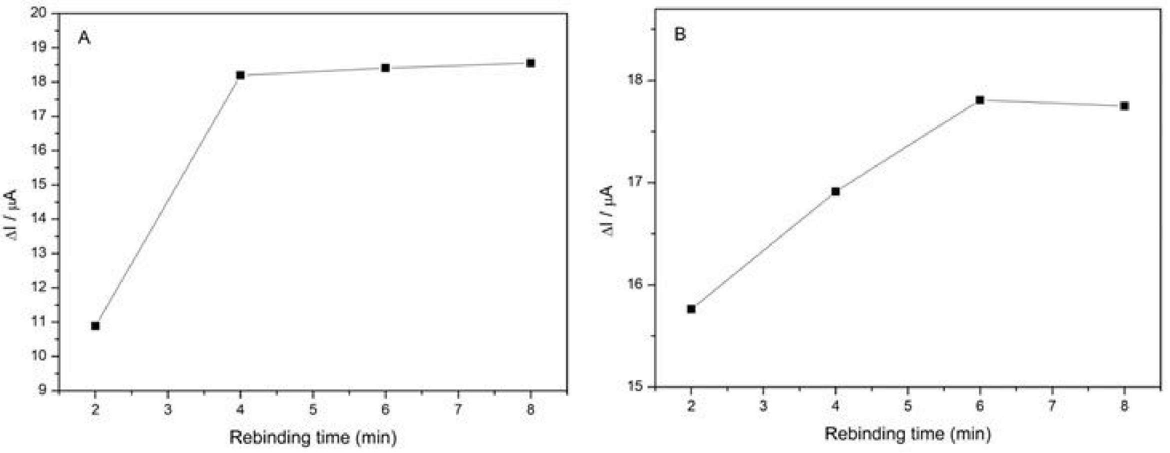

Figure S8. (A) Effect of rebinding time of D-xylose and (B) D-arabinose on peak current variation at the concentration of 10.0 x 10^{-3} mol L^{-1} $K_3[Fe(CN)_6]$ in 1.0 mol L^{-1} KCl.

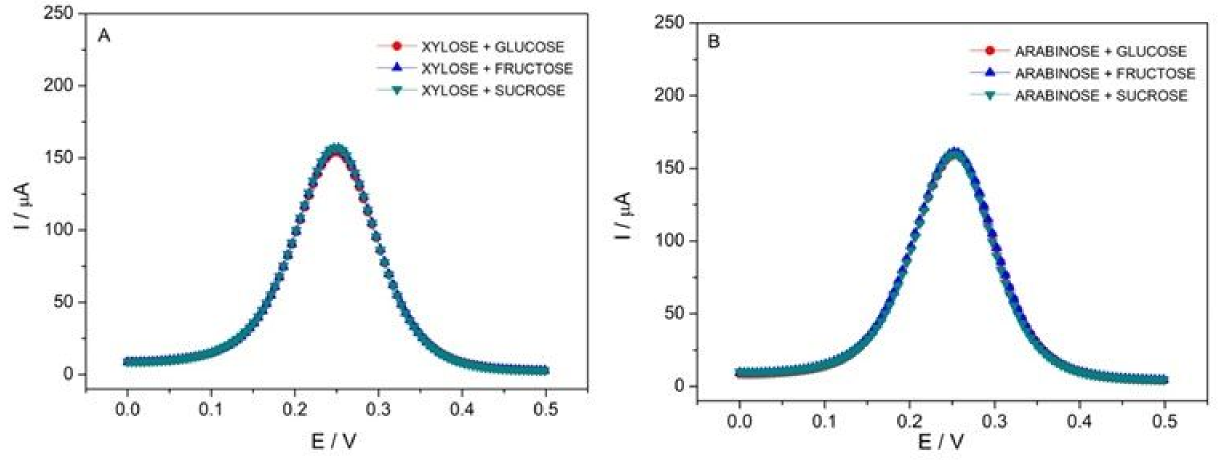

Figure S9. (A) Differential pulse voltammograms of sensor Xil-MIP/FMWCNTs/GPE on the probe after adsorption in a solution containing 1 x 10^{-11} D-xylose + 1 x 10^{-10} D-glucose, 1 x 10^{-11} D-xylose + 1 x 10^{-10} D-fructose and 1 x 10^{-11} D-xylose + 1 x 10^{-10} D-sucrose. (B) Differential impulse voltammograms of sensor Ara-MIP/FMWCNTs/GPE in the probe after adsorption on a solution containing 1 x 10^{-11} D-arabinose + 1 x 10^{-10} D-glucose, 1 x 10^{-11} D-arabinose + 1 x 10^{-10} D-fructose and 1 x 10^{-11} D-arabinose + 1 x 10^{-10} D-sucrose.

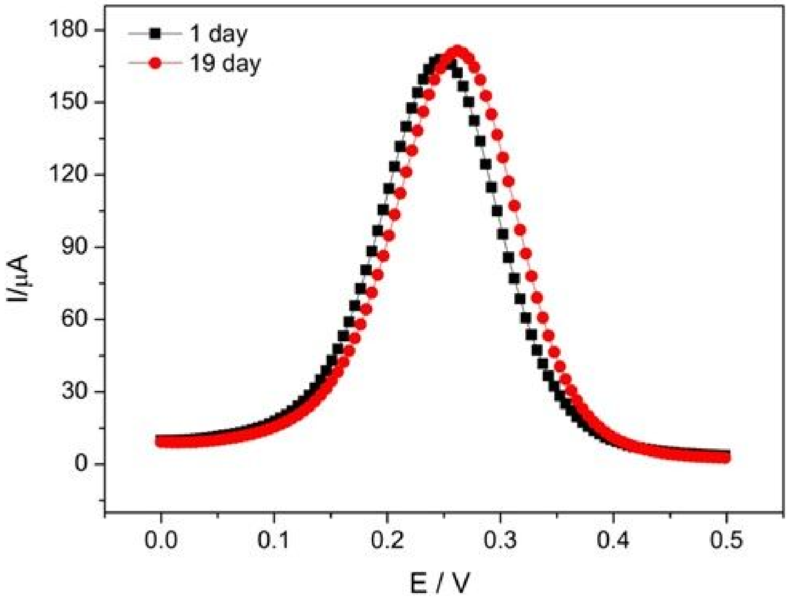

Figure S10. Differential pulse voltammograms for the sensor after one day and 19 days in the 10.0 x 10^{-3} mol L^{-1} $K_3[Fe(CN)_6]$ solution in 1.0 mol L^{-1} KCl.

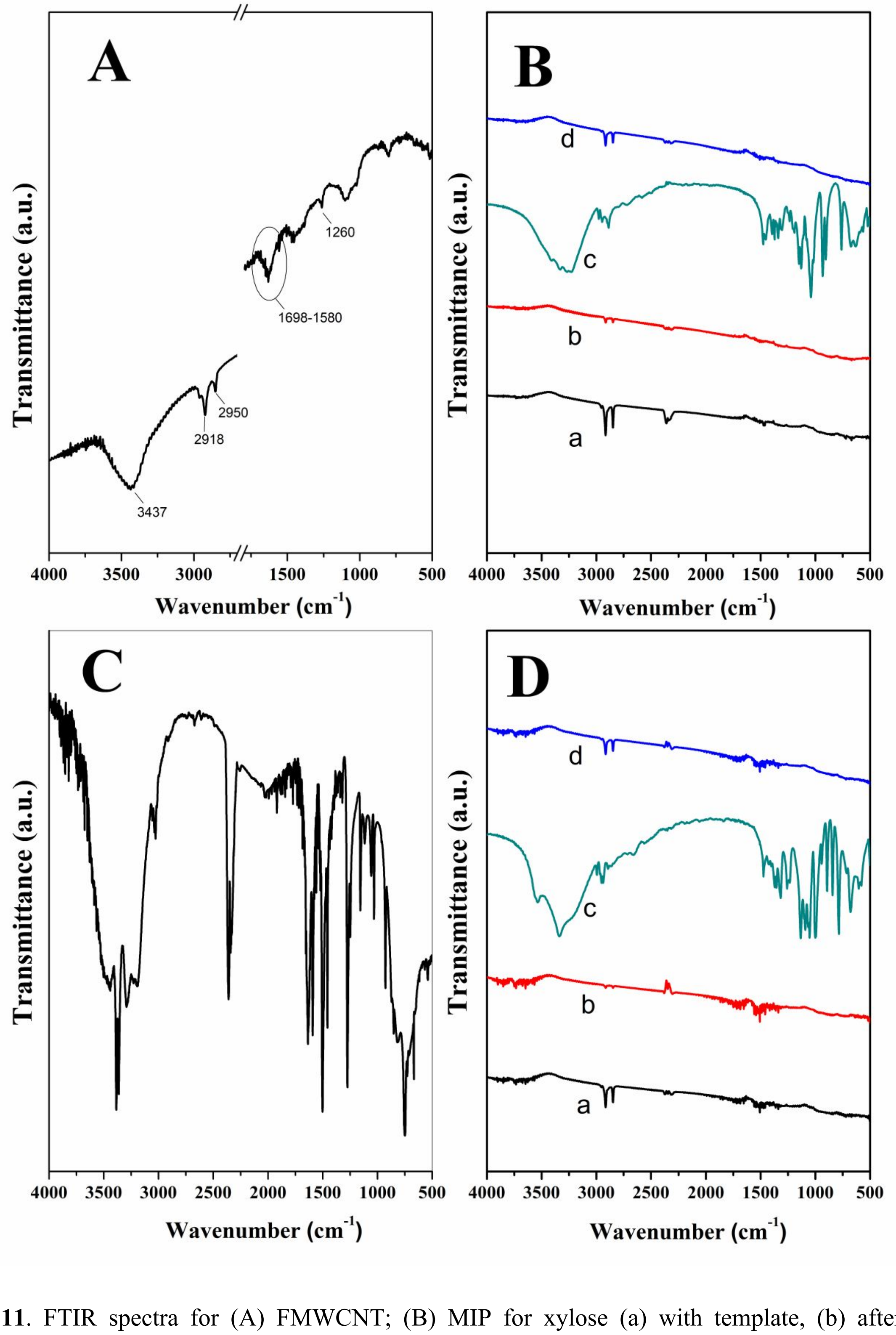

Figure S11. FTIR spectra for (A) FMWCNT; (B) MIP for xylose (a) with template, (b) after template extraction, (c) xylose and (d) after the rebinding process; (C) o-PD and (D) MIP for arabinose (a) with template, (b) after template extraction, (c) arabinose and (d) after the rebinding process.
